# Evaluation of traps and lures for mosquito vectors and xenomonitoring of *Wuchereria bancrofti* infection in a high prevalence Samoan Village

**DOI:** 10.1186/s13071-015-0886-2

**Published:** 2015-05-28

**Authors:** Limb K Hapairai, Catherine Plichart, Take Naseri, Ualesi Silva, Lameko Tesimale, Paulo Pemita, Hervé C Bossin, Thomas R Burkot, Scott A Ritchie, Patricia M Graves, Wayne Melrose, Hayley Joseph

**Affiliations:** Institut Louis Malardé, Papeete, French Polynesia; Ministry of Health, Apia, Samoa; Australian Institute of Tropical Health and Medicine, James Cook University, Cairns and Townsville, Australia; Current address: Walter and Eliza Hall Institute of Medical Research, 1G Royal Parade, Parkville, VIC 3053 Australia

**Keywords:** Filariasis, Transmission, *Aedes*, Elimination, Xenomonitoring, Samoa, BG-sentinel, Mosquito trap, Mosquito vector

## Abstract

**Background:**

Elimination of lymphatic filariasis (LF) in Samoa continues to be challenging despite multiple annual mass drug campaigns aimed at stopping transmission by reducing the prevalence and density of microfilaraemia. The persistence of transmission may be partly related to the highly efficient *Aedes* vectors. The assessment of pathogen transmission by mosquito vectors and of vector control relies on the ability to capture mosquitoes efficiently. The aims of this study are to compare trapping methods to capture LF-infected mosquitoes and determine the role in transmission of the species of *Aedes* mosquitoes in the area*.*

**Methods:**

Fasitoo-Tai village was the chosen site because of persistent transmission despite annual mass drug administration. Sampling methods included BioGents Sentinel (BGS) trap, human-baited collections (HBC) and the Centers for Disease Control (CDC) trap. BGS and CDC traps were baited with BG-lure, CO_2_, and/or octenol. Individual trap locations were geo-located and efficiency of sampling methods was evaluated using a randomized Latin-square design in two locations. Number of mosquitoes collected (male and female), as well as species for each trapping method were determined. Additionally, *Ae. polynesiensis* and *Ae.* (*Finlaya*) spp. females were pooled by trap method and analysed for filarial DNA. Infection prevalence was estimated using the PoolScreen software.

**Results:**

The BGS trap with any type of bait collected more mosquitoes compared to both the CDC trap and the HBC. The BGS trap baited with BG-lure collected more mosquitoes than with CO_2_ and octenol. There were no significant differences between trapping methods in terms of proportions of infected females collected. The prevalence of filarial infection in *Ae. polynesiensis* and *Ae.* (*Finlaya*) spp. was estimated at 4.7 % and 0.67 % respectively.

**Conclusions:**

This study supports the use of the BGS trap for research on and surveillance of the mosquito vectors of LF in Samoa. The BGS trap is a suitable and safer alternative to HBC for sampling *Ae. polynesiensis* and *Ae.* (*Finlaya*) spp., which continue to be the predominant vectors of LF. Of concern was the high prevalence of LF in mosquitoes despite a recent mass drug administration programme. This highlights the urgency for updated policies concerning filariasis elimination in Samoa.

## Background

*Wuchereria bancrofti* is the main causative parasite of lymphatic filariasis (LF), which afflicts over 120 million people, with 1.3 billion people at risk in 73 countries [1]. Samoa has been known as a highly LF endemic country since the 1920s, and has been conducting mass drug administration (MDA) campaigns intermittently since the 1960s to control the disease [2]. The first MDA campaigns used diethylcarbamazine (DEC) and were successful in reducing the prevalence of microfilariae (Mf) from 21 % in 1964 to 0.14 % in 1973 [3]. Unfortunately, a few years after cessation of MDA, a resurgence of LF occurred [4]. Prevalence of Mf was estimated at 4.3 % in 1993 [5]. It then fell to 1.1 % Mf prevalence (4.2 % by immunochromatographic test (ICT), N = 4054) after five rounds of MDA with DEC or DEC plus ivermectin between 1993 and 1997 [5]. In 1999, prevalence by ICT was still at 4.5 % in a larger survey of 7006 people in 27 villages [2].

The Global Programme to Eliminate Lymphatic Filariasis (GPELF), under the direction of the World Health Organization (WHO), was launched in the late 1990s and directed endemic countries to implement an annual single-dose MDA using a combination of anti-filarial drugs [6]. Under the auspices of the GPELF, the Pacific Programme for the Elimination of LF (PacELF) was launched in 1999 in 22 Pacific island countries and territories (PICTs) [7]. In Samoa as in other Pacific nations, the strategy to interrupt transmission was based solely on annual co-administration of single-dose DEC with albendazole.

Since the start of PacELF, Samoa completed five rounds of MDA from 1999 to 2003, and, after demonstration of persistent antigenaemia by ICT, two further MDA rounds were undertaken in 2006 and 2008 [8, 9]. Prevalence in all ages by ICT and Mf was estimated to be 2.6 % and 0.6 % respectively (N = 6448) in a nationwide survey in 2007 [10]. A follow-up study in five villages in 2008 (N = 2474) revealed pockets of residual prevalence ranging from 0 to 3.2 % for Mf and 1.6 to 14.6 % by ICT; the village with the highest prevalence was Fasitoo-Tai on Upolu island [11, 12]. As a consequence, an additional nationwide MDA occurred in November 2011 and two more rounds in part of the country are planned following a Transmission Assessment Survey in 2013 which demonstrated continuing transmission in north west Upolu [Government of Samoa Ministry of Health.: Lymphatic Filariasis Transmission Assessment Survey 2013. Final Report 11 June 2013, unpublished]. Although MDA temporarily decreases the density of Mf [13], the effectiveness of MDA in many PICTs is compromised by issues with MDA participation [14] and the biology of the vector [15].

The day-biting *Ae. polynesiensis* is the primary vector of LF in the Polynesian PICTs including Samoa [16]. MDA alone may not be sufficient to eliminate LF because of the biology of the vector which exhibits a negative density-dependence with the filarial parasite [15]. Paradoxically *Ae. polynesiensis* becomes a more efficient vector when microfilaraemia density is low, a scenario which can be created by MDA [17, 18].

Vector control integrated with MDA could facilitate LF elimination by breaking transmission in both human and vector cycles. However, the exophilic, semi-urban, and day-biting behaviour of *Ae. polynesiensis* [19] and its ability to utilise a variety of domestic and natural containers as larval habitat challenges many conventional vector control methods. Vector control for the secondary LF vector, *Ae. samoanus,* is also difficult as this breeds in *Pandanus* axils and bites throughout the night both indoors and outdoors [20]. The presence of both day and night biting LF vectors in Samoa increases the risk of LF transmission compared to many other LF endemic countries with only one major LF vector species. It is critical that novel methods of vector control be developed.

An essential pre-requisite to any vector intervention is the ability to monitor the mosquito population. Effective methods for sampling *Ae. polynesiensis* and *Ae. samoanus* must also be cost-effective to implement in Samoa. Previous research in Samoa involving mosquito sampling occurred prior to the early 1980s and the methods of choice were the human biting or landing catch (HLC) [20–23]. Obtaining ethical approval for the human landing catch in LF endemic areas is challenging because mosquitoes are allowed to land on the human collector, increasing the risk of mosquito bites and thus pathogen transmission. An alternative to the HLC is the human bait catch (HBC). The HBC uses aspirators to capture mosquitoes attracted to a human before they land. The HBC thus offers reduced risk to the collector from bites by infected mosquitoes, but both HLC and HBC are influenced by the attractiveness of the collector and are impractical to use in some areas, particularly where arbovirus transmission is ongoing. Traps such as the BG-Sentinel (BGS) trap (BioGents GmbH, Regensburg, Germany), provide a standardized method and may be used over extended geographical areas. BGS traps have been successfully used in American Samoa to capture *Ae. polynesiensis* [24–26] but BGS trapping for *Ae. samoanus* has been less well investigated. Moreover, previous studies have not compared the sampling efficiency of traps against the HBC.

The first objective of this study was to evaluate the efficacy of different methods to sample the vectors of LF in Samoa using traps with several different lures and/or carbon dioxide [26]. The second objective was to test the collected mosquitoes for LF by PCR [27] to estimate the level of LF infections in different vector species in the village in order to provide useful information to the LF control programme on vector surveillance methods and the usefulness of xenomonitoring.

## Methods

### Study area

**Fig. 1:**
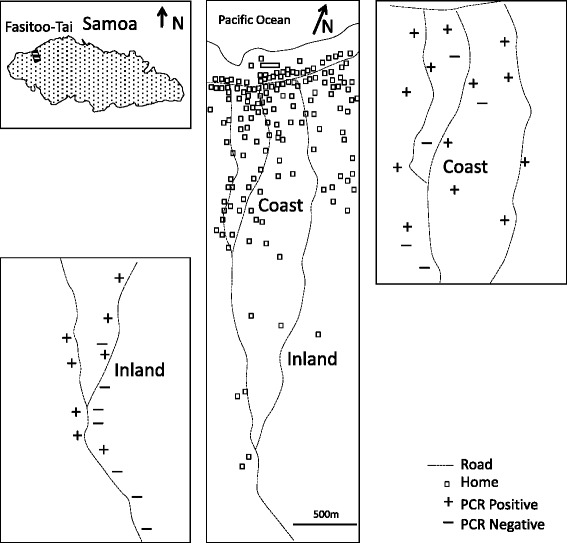
Location of Fasitoo-Tai, Samoa and xenomonitoring results. Locations of households in Fasitoo-Tai village and mosquito sampling sites with PCR positive (+) and negative (−) pools of female *Ae. polynesiensis* collected in traps (BGS and CDC) and HBC

The village of Fasitoo-Tai (approximately 6.8 km^2^) situated on the northwest coast of Upolu island (Fig. 1) was selected based on previous filariasis surveys indicating persistent filariasis transmission with high Mf prevalence [11, 12]. The study was undertaken in February 2012, 3 months after a nationwide MDA [Government of Samoa Ministry of Health: Report of Samoa mass drug administration for lymphatic filariasis conducted on 25–27 November 2011, unpublished].

Fasitoo-Tai is composed of two neighbourhoods, one coastal and another inland, separated by plantations and forests. Coastal dwellings are located more closely to one another than those more inland. Western style homes are common on the coast while only open Samoan ‘fale’ are observed inland. The main breeding containers were both natural (tree holes, rock hole, rat-chewed coconuts, cocoa pods) and domestic containers (tyres, small plastic containers, 50 gal drums, uncovered tanks).

GPS locations and elevations were measured with a Garmin 78S model (Garmin International, Inc., Olathe, KS) and referenced with Google Earth software. Replicates of the Latin-square trapping method comparison experiments described below were conducted at two coastal locations separated by 1.5 km (13°50'3.34"S 171°58'0.11"W and 25 m elevation) or inland (13°51'35.85"S 171°57'48.36"W and 120 m elevation).

### Sampling methods

Three collection methods were evaluated in a Latin Square design: the BGS, CDC trap and the HBC (Table 1):

**Table 1:** Description of sampling methods

Experiment	Number of replications	Number of repeats	Treatments	Attractant	Average collection time
Latin square BGS vs HBC	2	4	BGS + C/O	CO2, Octenol	24 h
	2	4	BGS + L	BG-lure	24 h
	2	4	BGS + L/C/O	BG-lure, CO2, Octenol	24 h
	2	4	HBC	Human	15 min
Latin square BGS vs CDC	2	3	CDC + C	UV-light, CO2	24 h
	2	3	CDC + C/O	UV-light, CO2, Octenol	24 h
	2	3	BGS + C/O	CO2, Octenol	24 h
Daytime HBC	27	N/A	HBC	Human	15 min
Night-time HBC	25	N/A	HBC	Human	30 min

HBC were conducted using the hand-held InsectaZooka™ (IZ) aspirator (Bioquip, Rancho Dominguez). While being much lighter in weight, the IZ aspirator has been shown to be equally efficient for collecting *Ae. polynesiensis* as the Centers for Disease Control and Prevention-Backpack (John Hock, Gainesville, FL) in HBC [28].

The BGS trap and UV LED CDC (CDC) trap (Bioquip, Rancho Dominguez, CA) were baited with BG-lure (a blend of lactic acid, ammonia, and caproic acid) or octenol lure (Bioquip, Rancho Dominguez, CA) in conjunction with yeast-produced CO_2_ [29].

Carbon dioxide was generated by mixing 35 g instant dry yeast - *Saccharomyces cerevisiae* (New Aule, Zhuhai Ziying Biotechnology CO., LTD, China), 700 g powdered sugar (Chelsea, Auckland, New Zealand) in 2.5 L tap water in 5 L plastic bottles [29]. CO_2_ was directed to the trap using polyvinyl chloride tubing connected at the bottom of the BGS trap or the top of the CDC trap to allow CO_2_ to diffuse from the trap. A study under similar climatic conditions demonstrated an average CO_2_ generation rate of 104 ml/min [30].

BGS and CDC traps were suspended from a tree branch allowing traps to suspend 20–30 cm above the ground. Engine grease was applied on the strings used for trap suspension, battery connection and on the CO_2_ tubing to prevent ant predation of collected mosquitoes. All traps and the IZ aspirator (CDC trap fitted with a 12 V-to-6 V converter) were powered using 12-V, 20 Ah batteries (Fullriver, Guang Zhou City, China). The average airflow for all collection methods was 13.0 m/s.

HBC involved a human acting as bait and an operator to capture all blood-seeking mosquitoes flying around the volunteer (≈50 cm). Operator and volunteer did not use repellent but wore long trousers and shirts to protect from mosquito bites.

### Sampling routine

A randomized 4 × 4 Latin Square experimental design was used to compare the BGS trap and HBC, (Table 1). In these experiments, BGS traps were baited with either BG-lure (BGS + L), CO_2_ plus octenol (BGS + C/O), or BG-lure with CO_2_ plus octenol (BGS + L/C/O) and compared to the HBC with the IZ aspirator. A total of 8 sites, 4 sites per neighbourhood, were used for the BGS trap vs HBC experiment.

A randomized 3 × 3 Latin Square experimental design compared the BGS and CDC traps (Table 1), in which the CDC trap with either CO_2_ (CDC + C), or CO_2_ plus octenol (CDC + C/O) was compared to the BGS trap with CO_2_ plus octenol (BGS + C/O). A total of 6 sites, 3 sites per neighbourhood, for each treatment were used for the BGS vs CDC experiment.

The distance between sites was greater than 1.5 km to limit competition between trapping methods. For both experiments, BGS and CDC traps were collected and rotated every 24 h within a neighbourhood (i.e., coast or inland) to minimize bias associated with collection location. Evaluations of BGS vs HBC were carried out between February 15^th^ to 21^th^, 2012 with HBC being done at 9:00 AM (coast) and 10:30 AM (inland). Evaluations of BGS vs CDC were carried out from February 21^th^ to 24^th^, 2012.

For the purpose of xenomonitoring evaluation, additional HBCs were performed during the daytime (10:00 AM-12:00 PM) and night-time (10:00 PM-12:00 AM) in various locations in the Fasitoo-Tai district from February 15^th^ to 29^th^, 2012. Night HBC were performed near *Pandanus* plants to increase the collection of *Ae.* (*Finlaya*) spp. Additional HBC collections were done approximately every 250 m between the coast (belt road) and inland (1 km away from the last home), to cover the clustering of LF cases [12].

### Mosquito identification

Mosquitoes were separated from other collected insects (mostly Tipulidae and Noctuidae) within 6 h after collection. All Culicidae were preserved on silica gel during transportation to the Institut Louis Malarde laboratory, French Polynesia for morphological identification. Identification of *Ae.* (*Finlaya*) to species was not possible due to unreliable dichotomous key characters (e.g., scales on wings from this group are used to distinguish between species in this group (*Ae. oceanicus, Ae. samoanus, and Ae. tutuilae*)) [31]. *Ae. polynesiensis* and *Ae.* (*Finlaya*) spp. female specimens were pooled (up to n = 20) in 1.5 ml tubes and preserved frozen for later analysis.

### Xenomonitoring

DNA from *Ae. polynesiensis* and *Ae.* (*Finlaya*) spp. females collected in Fasitoo-Tai was extracted in pools using the Qiagen DNeasy kit protocol (Qiagen, Hilden, Germany) as previously described [25]. Briefly, mosquitoes were placed in 2 mL grinding tubes and dried for one hour at 90 °C. Glass ball bearings were added with 180 μL ATL to the grinding tube and vortexed twice for 7.5 min (20/s) to macerate the specimen. The tubes were spun briefly, and 20 μL of proteinase K was added and vortexed before incubating for 30 min at 56 °C. The tube was again spun briefly, and 20 μL of proteinase K was added and vortexed before incubating for 30 min at 56 °C. After a brief spinning, 200 μL lysis buffer was added to the samples and vortexed for 15 s. The incubated material was then spun at maximum speed for 5 min and supernatant was transferred into a clean 1.5 ml tube and heated to 100 °C before being put on ice. In a clean 1.5 ml tube, 50 μL of 98 % ethanol and 100 μL of the supernatant was mixed and applied to the Qiagen DNeasy spin column. The column was washed twice with 500 μL buffer AW1 and once with 500 μL AW2. DNA was eluted from the column into a 1.5 ml tube by adding 125 μL of the AE buffer. This was repeated twice. Extracted DNA was used for qPCR assay using the *W. bancrofti* “LDR” repeat DNA primers [27] and SYBR Green fluorescence dye with melting analysis run on a Bio-Rad I-Cycler (Bio-Rad, Hercules, CA) [30]. DNA for positive controls was extracted from *W. bancrofti* and negative control from non-infected laboratory reared *Ae. polynesiensis* mosquitoes.

### Data analysis

Comparisons between numbers of captured mosquitoes of the same species were transformed as log_10_(x + 1) to correct for lack of normality and unequal variance in the raw data before Analysis of Variance (ANOVA) and pairwise comparisons by Tukey’s multiple comparisons test. LF infections in *Ae. polynesiensis* collected by different methods were analysed using a Chi-square goodness of fit test to compare the number of positive and negative PCR pools. Statistical analysis was carried out using GraphPad Prism version 5.0 (GraphPad Software Inc., La Jolla CA). The PoolScreen (v. 2.02) software (Department of Biostatistics and Division of Geographic Medicine, University of Alabama at Birmingham, USA) [32] was used to estimate *W. bancrofti* prevalence by maximum likelihood estimates (MLE) with 95 % confidence intervals based on likelihood ratio method.

### Ethics approval

This study was conducted under biosafety approval number SPHTMRS-2011-2, Institutional Biosafety Committee, James Cook University. The study protocol was also reviewed and approved by the Health Research Committee of the Samoan Ministry of Health on August 17^th^, 2011.

## Results

### BGS vs HBC

A total of 170 male and 3602 female *Ae. polynesiensis*, 54 male and 29 female *Ae. aegypti,* 58 male and 38 female *Ae. upolensis,* 93 male and 373 female *Culex quinquefasciatus*, and 3 male and 107 female *Ae.* (*Finlaya*) spp. were collected (Table 2).

**Table 2:** Mean (± standard error of the mean) female mosquitoes collected per trapper sampling period in Fasitoo-Tai using BGS, CDC and HBC sampling methods

Comparison 1: BGS vs HBC Latin Square				
Species^1^	HBC	BGS + C/O	BGS + L/C/O	BGS + L
*Ae. polynesiensis*	17.25 ± 2.17 a	85.0 ± 18.48 b	136.75 ± 20.32 bc	211.25 ± 44.35 c
*Ae.* (*Finlaya*) spp*.* ^*2*^	0.0 ± 0.0 a	5.75 ± 2.72 ac	5.25 ± 1.97 bc	4.38 ± 3.29 ac
*Ae. aegypti*	0.0 ± 0.0 a	0.88 ± 0.74 a	1.5 ± 1.0 a	1.25 ± 0.9 a
*Ae. upolensis*	1.25 ± 1.25 a	0.5 ± 0.27 a	1.25 ± 0.86 a	1.75 ± 0.77 a
*Cx. quinquefasciatus*	0.0 ± 0.0 a	16.25 ± 8.16 bc	28.63 ± 22.38 bc	1.75 ± 0.75 ac
Comparison 2: BGS vs CDC Latin Square				
Species^1^	CDC + C	CDC + C/O	BGS + C/O	
*Ae. polynesiensis*	1.50 ± 0.97 a	0.50 ± 0.43 a	69.83 ± 19.12 b	
*Ae.* (*Finlaya*) spp*.* ^*2*^	0.33 ± 0.18 a	1.17 ± 0.35 ab	5.5 ± 2.63 b	
*Ae. aegypti*	0.17 ± 0.14 a	0.0 ± 0.0 a	0.0 ± 0.0 a	
*Ae. upolensis*	0.0 ± 0.0 a	0.0 ± 0.0 a	0.33 ± 0.29 a	
*Cx. quinquefasciatus*	8.67 ± 3.19 a	4.67 ± 1.81 a	24.0 ± 15.99 a	
*Cx. annulirostris*	3.33 ± 1.88 a	0.17 ± 0.14 a	0.0 ± 0.0 a	

^1^For each species, means in the same row followed by the same letter are not significantly different (Tukey’s multiple comparison test, *P* = 0.05, on log_10_(x + 1) transformed catch)

^2^Includes species *Ae. oceanicus, Ae. samoanus*, or *Ae. tutuilae*

HBC: human bait catch; BGS: BG-Sentinel; CDC: Centers for Disease Control UV-light trap. Traps were baited with CO2 (C), octenol (O), and/or BG-lure (L)

BGS with any of the lures tested collected more male and female mosquitoes (except for *Ae.* (*Finlaya*) spp. males) than HBC (data for males not shown). Addition of the BG-lure to the BGS trap (BGS + L) resulted in collection of significantly more *Ae. polynesiensis* males (F = 5.56, df = 3, P = 0.004) and females (F = 32.03, df = 3, P < 0.0001) than the BGS + C/O. Addition of the BG-lure to BGS + C/O trap (BGS + L/C/O) increased female catches of *Ae. polynesiensis* and *Cx. quinquefasciatus* (F = 5.43, df = 3, P = 0.004). Statistical analysis for female *Ae*. (*Finlaya*) spp. (F = 3.26, df = 3, P = 0.035) and other species collected is presented in Table 2.

### BGS vs CDC

A total of 10 male and 431 female *Ae. polynesiensis*, one female *Ae. aegypti,* 19 male and 2 female *Ae. upolensis,* 41 female *Ae.* (*Finlaya*) spp., 82 male and 224 female *Cx. quinquefasciatus* and 3 male and 21 female *Culex annulirostris* were collected (Table 2).

BGS + C/O collected both more male and female mosquitoes than the CDC + C or CDC + C/O except for male and female *Cx. annulirostris* and male *Ae.* (*Finlaya*) spp. The BGS + C/O traps collected more female *Ae. polynesiensis* (F = 37.86, df = 2, P < 0.0001) and female *Ae.* (*Finlaya*) spp*.* (F = 5.362, df = 2, P = 0.017), than the CDC + C or CDC + C/O.

### Additional HBC

Twenty seven additional daytime HBC collections (ca. 15 min each) were performed for an average catch per collection of 1.04 male and 13.28 female *Ae. polynesiensis*, 0.48 male and 0.12 female *Ae. aegypti,* 2.64 male and 2.28 female *Ae. upolensis,* 0.04 males and 0.04 female *Cx. quinquefasciatus*. No *Ae.* (*Finlaya*) spp. were collected during daytime HBC.

From the 25 additional night-time HBC (ca. 30 min each) conducted, the average number of mosquitoes per HBC was 7.77 female *Ae. polynesiensis,* 1.33 female *Ae.* (*Finlaya*) spp.*,* 0.11 males and 0.66 female *Cx. quinquefasciatus.* Only 9 collections were positive for *Ae*. (*Finlaya*) spp.

### Species distribution

*Ae. aegypti* was collected only in the coastal neighbourhood where homes were more abundant and densely packed. Other species were collected in both coastal and inland neighbourhoods.

### Xenomonitoring

A total of 144 pools of female mosquitoes were analysed by PCR (Table 3). For *Ae. polynesiensis,* a total of 127 pools (mean = 17.56; range = 4–20) were analysed of which 74 pools (58.3 %) were positive for LF. A similar proportion of LF positive pools was obtained irrespective of the sampling method used, and there was no significant difference between the proportion of infected pools of mosquitoes from HBC, BGS + C/O, BGS + L/C/O, and BGS + L (*X*^*2*^ = 0.61, df = 3, P = 0.89). From all the pools combined, the overall prediction of prevalence based on PoolScreen software was 4.7 % (95 % Confidence interval (CI) 3.6 % to 6.1 %), (Table 3). For *Ae.* (*Finlaya*) spp., 17 pools (mean = 8.06; range = 2-20) from all collected females (i.e., from both Latin square comparisons and additional HBC) were analysed of which one pool (5.8 %) was positive. The predicted LF prevalence in this group of species was 0.67 % (95 % CI 0.002 % to 3.4 %), (Table 3).

**Table 3:** Summary of xenomonitoring by PCR of female *Ae. polynesiensis* and *Ae.* (*Finlaya*) spp. sampled in Fasitoo-Tai using various collection methods

Species	Collection method	LF positive pools	LF negative pools	% pools LF positive	No. mosquitoes analyzed	Average pool size	% Predicted LF prevalence in mosquitoes (95 % CI)
*Ae. polynesiensis* (Comparison 1)	HBC	6	4	60.0 %	135		
	BGS + C/O	16	11	59.3 %	503		
	BGS + L/C/O	21	11	65.6 %	640		
	BGS + L	18	14	56.3 %	621		
*Ae. polynesiensis* (additional HBC)	HBC	13	13	50.0 %	332		
TOTAL							
*Ae. polynesiensis*	All	4	53	58.3 %	2251	17	4.7 % (3.6 to 6.1 %)
*Ae.* (*Finlaya*) spp*.* ^1^	All^2^	1	16	5.9 %	153	9	0.67 % (0.21 to 0.34 %)

^1^Includes species *Ae. oceanicus, Ae. samoanus*, and/or *Ae. tutuilae*

^2^Females collected in BGS/HBC and BGS/CDC comparisons and additional HBC

## Discussion

This study compares three sampling methods for collecting adult *Ae. polynesiensis* and *Ae*. (*Finlaya*) spp. in a Samoan village. For both male and female *Ae. polynesiensis*, the BGS is a suitable alternative to HBC in Samoa. Irrespective of the bait used, BGS collected more mosquitoes than HBC.

The trapping method with the highest yield for day-time *Ae. polynesiensis* collection was the BGS + L. Addition of CO_2_ plus octenol to BGS traps did not significantly increase the number of *Ae. polynesiensis* males or females compared to BGS + L traps. For males, addition of the BG-lure increased the number of specimens collected. These results confirm previous trap studies conducted in French Polynesia [30]. It is important to note that the BGS trap + C/O also collected significantly more female *Ae. polynesiensis* than the CDC trap + C/O.

For *Ae.* (*Finlaya*) spp*.*, the BGS + C/O collected more mosquitoes than other trapping methods in both comparisons. Schmaedick et al. [24] observed that CDC light traps baited with CO_2_ collected more specimens of this species than BGS + L. Our study shows that addition of octenol to a UV LED CDC trap baited with CO_2_ increases its attractiveness for *Ae.* (*Finlaya*) spp. However, BGS + C/O traps collected more specimens than CDC + C or CDC + C/O traps suggesting it is better suited for sampling *Ae.* (*Finlaya*) spp. While the BGS + C/O collected fewer mosquitoes on average with means of 5.8 and 5.5 mosquitoes per collection (comparisons: BGS vs HBC and BGS vs CDC respectively) compared to 7.7 mosquitoes for night-time HBC, all BGS + C/O contained at least one *Ae.* (*Finlaya*) spp. compared to only 9 out of 25 samples for HBC.

The current research was limited by the inability to distinguish mosquito species for the *Ae*. (*Finlaya*) spp. based on morphology [31] because of wing damage sustained during the trapping. Genetic tools may be developed to distinguish these species but are costly. Instead, further development of future vector surveillance traps should be able to provide reliable catch rates while preserving features (i.e., scales) for downstream identification. Prior successful field collections of adult *Ae. samoanus* [20, 33] used human landing catches. Allowing mosquitoes to probe greatly reduced mobility thus permitting collectors to aspirate mosquitoes undamaged. Damaged wings in this study are most likely the result of continuous suction over longer periods with BGS and CDC traps over 24 h, and HBC night time collection lasting 30 mins respectively. Recent design development and validation of the Passive Box Trap for Kunjin vectors [34] may provide an alternative collection method for *Ae. samoanus.* This trap does not utilise suction power and captures mosquitoes as they follow CO_2_ up a pipe, but leads to a clear chamber where mosquitoes may be kept alive on honey for later harvest.

A recently published study revealed the first evidence of spatial clustering of LF in people in Fasitoo-Tai [12]. This research suggested that parasite transmission is focal and hence targeted vector control interventions could play a role in LF elimination. Investigation of clustering of infections in mosquitoes is beyond the scope of this study due to insufficient trap locations and high positivity rate in each trap, but deserves further investigation in relation to clustering of human infections. Given both human and mosquito population movement, an integrated and widespread vector control strategy coupled with closely monitored and high coverage MDA is likely to be required to achieve elimination.

The infection rate estimated at 4.7 % by PCR for *Ae. polynesiensis* in this study was high compared to 0.69 % and 0.28 % observed in neighbouring American Samoa [25, 26] and to 0.17 % and 0.44 % in Moorea, French Polynesia [35]. Previously recorded infection rates from dissected *Ae. polynesiensis* mosquitoes in Samoa were 8.35 % before and 0.61 % after MDA in 1965–1966 [36]. In the current study, for *Ae polynesiensis*, there was no difference in estimated infection rates between different trapping methods i.e., BGS traps and HBC. Thus the BGS offers a suitable alternative to the HBC as a tool for monitoring LF infection rates in areas where the vector for LF is *Ae. polynesiensis*.

The infection rate from PCR detection in *Ae*. (*Finlaya*) spp. was estimated at 0.67 % in our study, a much higher rate than in previous studies investigating *Ae. samoanus* by dissection [21, 36]. Yet, from the three species present in the *Ae.* (*Finlaya*) spp. group in Samoa, *Ae. samoanus* is the only LF vector described, and thus the infection rate for this species in Fasitoo-Tai is most likely underestimated.

The high rate of infection in both vector species was particularly unexpected considering the recent MDA in Samoa, including Fasitoo-Tai, which should have reduced microfilaraemia density and prevalence. One possibility is that participation in MDA remains low in Samoa [14]. The results indicate that future studies should use smaller pool sizes to obtain more precise estimates of prevalence of LF in the mosquito. Previous studies demonstrated the capability for the “LDR” repeat DNA primers to detect filarial DNA present in *Ae. polynesiensis* in low prevalence areas [25, 26, 35]. Xenomonitoring using this method is limited, particularly in high prevalence areas where the “LDR” primers may detect filarial DNA in the mosquito that is persistent after feeding on infected human carriers [37]. Although stage-specific assays have been developed for detection of infective mosquitoes [38, 39], these assays require validation in *Ae. polynesiensis* and *Ae. samoanus*. Further molecular investigations and validation of stage-specific assays for both vector species would provide a more accurate description of the transmission dynamics.

*Ae. polynesiensis* is the main vector of LF in Fiji, French Polynesia, Samoa and American Samoa. The population from these four PICTs (1,403,433) represents 60 % of the total population of PICT (2,340,647, excluding PNG) identified as endemic for LF and undergoing LF elimination programmes. For this reason, *Ae. polynesiensis* (especially when other secondary vectors are also present), poses a significant challenge to LF elimination for the Pacific region [15]; perhaps second only in importance to the challenge of residents who consistently are not offered or do not participate in MDA. Our findings highlight the strong urgency for agencies to coordinate and continue effective MDA where needed, and the need for supplemental effective vector control.

## Conclusions

Our study contributes to demonstrating the most effective vector sampling tool for future vector control programs in Samoa that is also relevant to other countries with *Ae. polynesiensis* as a vector, as well as providing updated information on the vector species present and their infection rates in an area of Samoa where transmission occurs. BGS traps offer a safe alternative to the previous gold standard of HBC. Additionally, *Ae. polynesiensis* was found to be the predominant vector, due to its greater abundance and an infection prevalence estimated at 4.7 % compared to 0.67 % for *Ae.* (*Finlaya*) spp. This alarmingly high prevalence, despite the recent MDA, focuses attention on the challenge of low treatment coverage of populations with DEC and albendazole during the MDA campaigns. This highlights the need for more stringent MDA with directly observed treatment and record keeping as well as the inclusion of vector control methods in high prevalence villages in Samoa.
